# Editorial: *Chlamydia-*host interaction and its pathogenic mechanism

**DOI:** 10.3389/fcimb.2024.1372714

**Published:** 2024-02-06

**Authors:** Zhou Zhou, Yuanjun Liu, Chunfu Yang, Hector Alex Saka

**Affiliations:** ^1^ Institute of Pathogenic Biology, Hunan Provincial Key Laboratory for Special Pathogens Prevention and Control, Hengyang Medical College, University of South China, Hengyan, China; ^2^ Department of Dermatovenereology, Tianjin Medical University General Hospital, Tianjin, China; ^3^ School of Public Health and Emergency Management, Southern University of Science and Technology, Shenzhen, China; ^4^ Laboratory of Cellular and Molecular Microbiology, Centre for Research in Clinical Biochemistry and Immunology (CIBICI), National Council on Scientific and Technical Research (CONICET), Cordoba, Argentina; ^5^ Department of Clinical Biochemistry, School of Chemical Sciences, National University of Cordoba, Cordoba, Argentina

**Keywords:** intracellular pathogens, bacterial pathogenesis, *Chlamydia*, *Chlamydia*-host interactions, Chlamydial disease

The order *Chlamydiales* currently includes four validly published families: *Chlamydiaceae*, *Parachlamydiaceae*, *Simkaniaceae*, and *Waddliaceae* (LPSN, 2024a). These four families encompass obligate intracellular Gram-negative bacteria that infect eukaryotic cells, from animals and insects to protozoa. All the *Chlamydiales* share a highly efficient propagation cycle that involves the alternation between two distinct forms: the infectious, dormant, and environmentally stable elementary bodies (EBs) and the replicative, labile, and non-infectious reticulate bodies (RBs). Once the EBs infect their host cells, they are internalized into a parasitophorous vacuole named an “inclusion” and then differentiate into RBs, which rely on nutrients acquisition from the host to replicate. At mid to late times post-infection, RBs redifferentiate into infectious EBs and exit to the extracellular environment where neighboring cells can be infected (Abdelrahman and Belland, 2005; Saka and Valdivia, 2010; Bayramova et al., 2018). Among the *Chlamydiaceae* family, the genus *Chlamydia* contains 13 species (LPSN, 2024b), 3 of which are the most relevant as human pathogens. *Chlamydia trachomatis* is the main bacterial cause of sexually transmitted infections and the leading agent of infectious blindness worldwide (WHO, 2018; WHO, 2022), *C. pneumoniae* is a common etiology of atypical pneumoniae (Aliberti et al., 2021), and *C. psittaci* causes psittacosis, a globally distributed and potentially fatal zoonotic disease transmitted to humans mainly by exposure to birds (Dembek et al., 2023; Liu et al., 2023).

Despite being major human pathogens, many unknowns remain regarding the molecular basis of *Chlamydia*–host interactions, primarily due to a longstanding genetic intractability that began to significantly change only about a decade ago [(Valdivia and Bastidas, 2018; Kedzior and Bastidas, 2019; Wolf et al., 2019; Fisher and Beare, 2023) and references therein]. The elucidation of how these microorganisms interact with host cells involves the dissection of different key steps in chlamydial virulence, including (but not limited to) *Chlamydia* adherence, entry, EB to RB differentiation and back, nutrient acquisition, chlamydial persistence, exit from infected cells, host cell pathway manipulations, immune response and evasion, and the specific role of each virulence factor in pathogenesis.

In this Research Topic, four manuscripts focus on different aspects of *Chlamydia*–host interactions and virulence.

Scanlon et al. studied the role of type III secretion system effector TmeB, which functions in *C. trachomatis* entry to epithelial cells. TmeB is encoded in a bi-cistronic operon together with TmeA, and they both are secreted within minutes after *Chlamydia* attachment. These authors generated and manipulated *ΔtmeA*, Δ*tmeB*, and Δ*tmeAB* mutants as well as strains null for multiple genes to uncover that, as opposed to TmeA, lack of TmeB does not significantly impair the invasion efficiency of *C. trachomatis.* Intriguingly, they found that loss of TmeB turned TmeA dispensable for invasion and that overabundance of TmeB hinders host cell invasion and Arp2/3-mediated actin polymerization. Altogether, these results point out a complex and dynamic interplay between TmeA and TmeB and host actin polymerization during chlamydial entry.

Olivera et al. investigated the interaction between *C. trachomatis* and human *Papillomavirus* (HPV) in an *in vitro* model mimicking a co-infection scenario with these two highly prevalent agents of sexually transmitted infections. These investigators found that infection of a human epithelial C33-A cell line expressing the major oncoproteins E6 and E7 from high-risk HPV-16 (E6E7 cells) resulted in upregulation of E6E7 and host cell inhibitory molecules PD-L1, HVEM, and CD160. Moreover, they found that *C. trachomatis* inclusions were smaller and produced lesser amounts of infectious progeny in E6E7 cells, in agreement with an electron microscopy analysis showing increased numbers of RBs and decreased EBs at late time points post-infection. These results imply that HPV and *C. trachomatis* may influence each other in a co-infection scenario and lead to enhanced oncogenicity and immunosuppression, highlighting the relevance of screening for the mutual infection to assess potentially bad outcomes in reproductive health.

Yao et al. studied a clinical case of psittacosis in an HIV patient suffering acute pneumonia. Interestingly, these authors were able to successfully use metagenomic next-generation sequencing (mNGS) combined with nested PCR and real-time PCR to confirm a diagnosis that is challenging for microbiology laboratories, especially in chronically ill patients. This report shows that mNGS is a promising new tool that, in combination with other tests, allows for rapid and accurate diagnosis and treatment of infections with fastidious pathogens including *C. psittaci*.

Turman et al. contributed a very interesting review article addressing the role of the conserved *Chlamydia* plasmid in virulence. The authors evaluated different published studies about genital, ocular, and gastrointestinal infection with *C. trachomatis* and *C. muridarum*, particularly focused on the potential role of the chlamydial plasmid in pathogenesis and disease development, infectivity, and inflammation as well as tissue- and species-specific differences. This review highlights that many questions about how the *Chlamydia* plasmid participates in virulence remain unanswered, pointing out that investigations oriented to addressing this matter are desirable to improve our knowledge gaps about how *Chlamydia* causes human disease.

In conclusion, this Research Topic contributes valuable information and original results on the difficult-to-study field of *Chlamydia* interactions with their hosts. Understanding the molecular aspects of how these elusive microorganisms propagate and cause disease is a prerequisite for the development of infection control strategies, including the formulation of effective anti-chlamydial vaccines.

## Author contributions

ZZ: Writing – review & editing. YL: Writing – review & editing. CY: Writing – review & editing. HS: Conceptualization, Writing – original draft, Writing – review & editing.
